# The vagus nerve: a cornerstone for mental health and performance optimization in recreation and elite sports

**DOI:** 10.3389/fpsyg.2025.1639866

**Published:** 2025-07-11

**Authors:** Christian Lopez Blanco, William J. Tyler

**Affiliations:** ^1^Department of Biomedical Engineering, Center for Neuroengineering and Brain Computer Interfaces, University of Alabama at Birmingham, Birmingham, AL, United States; ^2^Department of Occupational Therapy, School of Health Professions, University of Alabama at Birmingham, Birmingham, AL, United States

**Keywords:** vagus nerve, performance, cognition, stress, autonomic nervous system, sports, recovery

## Abstract

Decades of physiological and psychological research into human performance and wellness have established a critical role for vagus nerve signaling in peak physical and cognitive performance. We outline models and perspectives that have emerged through neuroscience and psychophysiology studies to elucidate how the vagus nerve governs human performance through its influence on central nervous system functions and autonomic nervous system activity. These functions include the monitoring and regulation of cardio-respiratory activity, emotional responses, inflammation and physical recovery, cognitive control, stress resilience, and team cohesion. We briefly review some useful interventions such as transcutaneous auricular vagus nerve stimulation, heart-rate variability biofeedback, and controlled breathing as accessible tools for enhancing vagal tone, improving executive functioning under pressure, and mitigating fatigue and burnout. We describe how these approaches and their biological underpinnings are rooted by psychological models like the Yerkes-Dodson law and Polyvagal theory to contextualize their effects on athletic performance. These perspectives suppor recent shifts in sports science toward integrating vagal-centered approaches as scalable, evidence-based strategies that can enhance human performance and wellness.

## Introduction

Optimal human performance depends on a finely tuned balance between the sympathetic *fight-or-flight* system and the parasympathetic *rest-and-digest* brake. This balance is regulated by the 10^th^ cranial nerve (CN X), also commonly known as the vagus nerve. This peripheral nerve provides rapid, bidirectional (afferent and efferent) communication between brain-stem nuclei and other vital organs, including the heart, lungs, spleen, and liver, as well as the small intestines. The structure and the function of the vagus enables swift cardiovascular down-regulation and continuous visceral feedback to the cortex (Berthoud and Neuhuber, 2000). Some visceral sensory functions of the vagus nerve underlie what many refer to as a sixth sense (Zagon, 2001; Zhao et al., 2022). Vagal regulation of cardiac activity is perhaps best recognized through the iconic mammalian diving reflex, which occurs when sensory fibers of the vagus and trigeminal nerve are stimulated by facial submersion to trigger bradycardia or a reduction in heart rate (Gooden, 1994; Khurana et al., 1980; Andersen, 1963). In this perspective article we briefly describe the anatomy and physiology of the vagus nerve in the context of recent evidence and neurobiological models to illustrate the essential roles of vagal activity in human performance and wellness.

High resting vagal tone, typically indexed by high-frequency heart-rate variability (HRV), is associated with lower resting heart rate (HR), more efficient baroreflexes, and greater neuro-visceral flexibility (Krygier et al., 2013). Because elite and recreational athletes face repeated exposures to heavy training loads and acute competitive stress, they offer a compelling model for translating vagal physiology into practice. Intense or poorly regulated arousal can erode mental health, slow cognitive processing, and prolong recovery (McLaughlin et al., 2013; Laborde et al., 2018a), whereas acute elevations in vagal activity, achieved through slow-paced breathing, HRV biofeedback, or transcutaneous auricular vagus-nerve stimulation (taVNS), have been linked to faster post-exercise heart-rate recovery, sharper executive function under pressure, and improved cognitive resilience (Çalι et al., 2023; Jacobs et al., 2015; Murphy et al., 2023). Through ascending pathways, taVNS is known to modulate activity of the locus coeruleus (LC) and norepinephrine (NE), which are involved in regulating *fight-or-flight* sympathetic responses, cortical arousal, and attention (Urbin et al., 2021; Sharon et al., 2020; Frangos et al., 2015). Through the descending cholinergic anti-inflammatory pathway, it has been shown to reduce the production of pro-inflammatory cytokines (Czura and Tracey, 2005; Pavlov and Tracey, 2012, 2022). With respect to athletic performance, a recent randomized trial demonstrated that a single week of daily taVNS increased maximal oxygen uptake and blunted exercise-induced inflammation in healthy adults (Ackland et al., 2025). As further detailed below, taVNS provides a means of modulating the autonomic nervous system to enhance physiological and psychological resilience.

**Figure 1:**
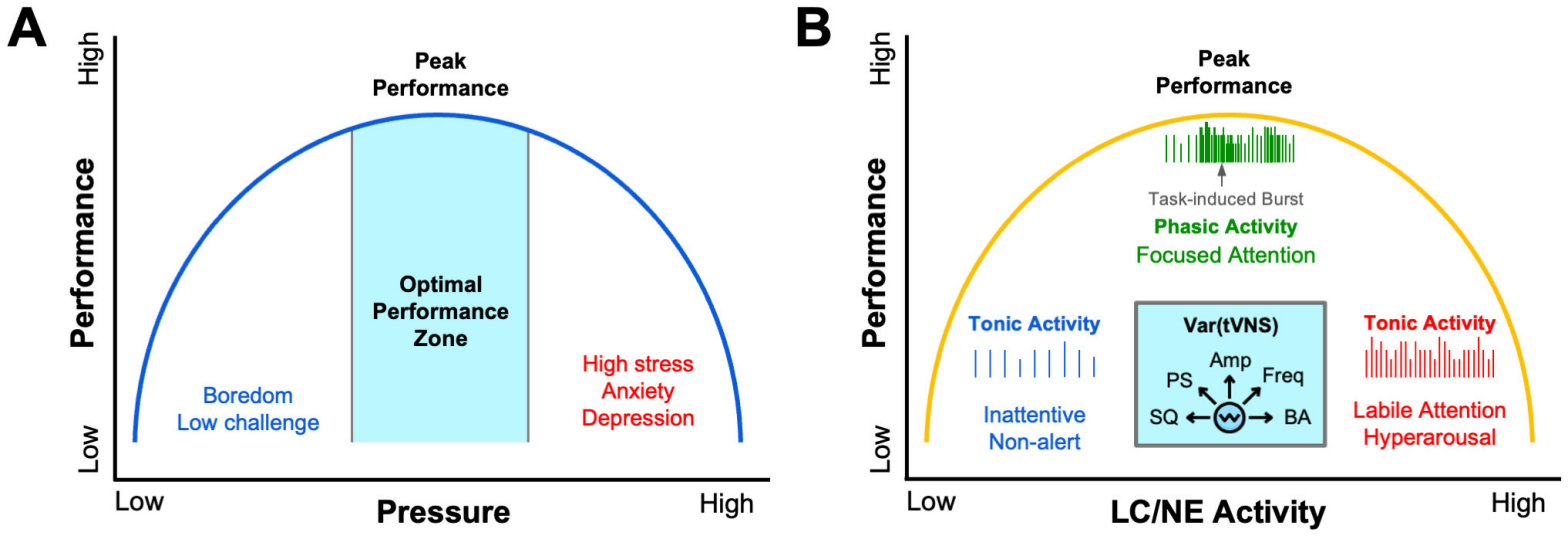
Psychophysiological models of human performance. Several models describing the effects of psychological and physiological variables on performance can be described by an inverted-U function. **(A)** The illustration is an adaptation of the canonical Yerkes-Dodson law, where performance is regulated as an inverted-U function of pressure, stress, or sympathetic arousal (Yerkes and Dodson, 1908). **(B)** Task-based performance can also be described by an inverted-U function of locus coeruleus (LC) and norepinephrine (NE) activity. It has been shown that low-frequency tonic activity of LC neurons occurs during periods of inattentiveness, while higher frequency tonic activity of LC neurons occurs during periods of hyperarousal when attention is labile (Aston-Jones and Cohen, 2005). As illustrated, during periods of peak task-based performance, LC neurons fire in a phasic manner (Aston-Jones and Cohen, 2005). Other data convincingly show that transcutaneous vagus nerve stimulation (tVNS) can tune arousal by modulating LC/NE activity across this performance curve as a function of several different stimulus variables (*cyan inset*). These tVNS variables that differentially influence autonomic arousal and LC/NE activity include: Stimulus Quality (SQ) including electrode coupling methods, human factors, stimulus comfort, and sensory intensity; Pulse Shape (PS) parameters such as biphasic, monophasic, interphase gap, pulse width, pulse symmetry and charge balance; Stimulus Amplitude and Current Density (Amp); Stimulus Frequency (Freq) ranging from low Hz to tens of kHz; and Baseline Arousal (BA) such as stress, physical or cognitive fatigue, and level of engagement.

Through the lens of psychophysiological models like Polyvagal theory, a deeper perspective of how vagus activity alters performance unfolds by distinguishing between an evolutionarily older, unmyelinated dorsal vagal pathway that mediates shutdown responses and a newer myelinated ventral branch that supports social engagement and rapid cardiac control (Porges, 2001). Athletes who can flexibly engage the dorsal *vagal brake* appear better able to operate within the optimal performance zone of the Yerkes–Dodson performance curve, alert yet composed, thereby avoiding the performance-sapping extremes of under- and over-arousal (Figure 1A) (Porges, 2009; Yerkes and Dodson, 1908). Mirroring the Yerkes-Dodson law, an inverted-U curve has also been used to model the influence of stress and LC/NE activity on task-based attention and performance (Figure 1B). It has been shown neurons of the LC tonically fire at low frequencies when a subject is bored or has low levels of engagement, while they fire tonically at high frequencies when attention is labile or subjects are hyper-aroused and easily distracted (Aston-Jones et al., 1999; Aston-Jones and Cohen, 2005). When subjects are optimally attentive and in a state of peak performance, neurons of the LC fire in a phasic mode reflecting task-based engagement (Aston-Jones et al., 1999; Aston-Jones and Cohen, 2005) (Figure 1B). We describe the implications of these models for understanding the role of vagal activity in sports performance, cognition, and mental health below.

Although interest in vagal regulation is growing, much of the existing literature remains fragmented, as most investigations address isolated outcomes such as cognition, inflammation, or team cohesion rather than offering an integrative model. Below, we provide a perspective that summarizes the latest neuro-cardiac research to describe vagal anatomy, physiology, and measurement in athletic contexts. We further provide a description of mechanistic links between vagal tone, cognitive control, emotion regulation, and recovery. Our perspective includes evidence for breathwork, HRV biofeedback, transcutaneous vagus nerve stimulation (tVNS), and environmental stressors acting as performance enhancers through modulation of vagal activity. By framing athletic readiness as a function of vagal tone and cardiac vagal activity, our perspective aims to equip coaches, clinicians, and sport scientists with empirically grounded approaches to optimize the performance of both mind and body.

## Vagal physiology and autonomic regulation

**Figure 2:**
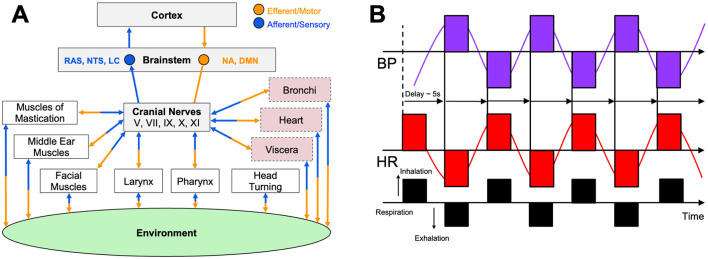
Central and peripheral mechanisms of autonomic arousal and the vagal brake. **(A)** The schematic shows the critical relationship between the environment, mind, and body as mediated by several cranial nerves, including the vagus (CN X), as similarly described by Polyvagal theory (Porges, 2009). The figure shows afferent (*blue*) and efferent (*orange*) connectivity between muscles and organs relay to several brainstem regions including the nucleus ambiguous (NA), the dorsal motor nucleus (DMN), and regions of the reticular activating system (RAS) like the locus coeruleus (LC) and nucleus of the solitary tract (NTS). Cranial nerve activity is transmitted to cardiorespiratory and upper-airway effectors while receiving afferent feedback that ultimately reaches cortical centers. These pathways are responsible for the real-time tuning of autonomic nervous system responses, including heart rate, respiration rate, and emotional reactivity to internal visceral states and environmental stimuli. **(B)** The figure illustrates how resonance-frequency breathing (*black*; ≈ 6 breaths · min^−^1) produces large, phase-locked oscillations in heart rate (HR; *red*) and blood pressure (BP; *purple*) separated by a ≈ 5 second baroreflex delay. This exemplifies maximal engagement of the vagal brake and high-amplitude heart-rate variability. **(A)** Is modified from Porges (2007) and **(B)** is adapted from Shaffer and Meehan (2020).

The vagus nerve (CN X), the body's largest parasympathetic conduit, contains mixed afferent and efferent fibers that emerge from the brain stem's nucleus ambiguus and dorsal motor nucleus, traverse the neck, and innervate the heart, lungs, and abdominal viscera (Berntson et al., 1993). Through acetylcholine release at the sinoatrial node, vagal efferent fibers slow the heartbeat and lower blood pressure, providing the rapidly adjustable vagal brake that counter-balances sympathetic drive and preserves homeostasis (Butt et al., 2019). At rest, strong vagal output produces a low resting heart rate and high beat-to-beat variability, or HRV, signaling an adaptable autonomic system (Carnevali and Sgoifo, 2014). When vagal tone is weak, sympathetic dominance emerges, promoting stress, hyper-arousal, and metabolic cost (McLaughlin et al., 2013). Vagal afferents simultaneously return visceral and baroreceptor signals to the brain stem and insula; this circuitry also triggers the cholinergic anti-inflammatory reflex, limiting cytokine release after physical or psychological stress (Berntson et al., 1993; Sloan et al., 1994). The organization of this system is captured in Figure 2A, which illustrates how the brainstem distributes vagal efferent signals to target organs, including the heart, pharynx, and bronchi, while concurrently receiving afferent input from baroreceptors and visceral tissues that shape brain and behavioral responses.

Stephen Porges' polyvagal theory refines this picture by describing two distinct vagal subsystems: an evolutionarily older, unmyelinated dorsal branch mediating shutdown and an evolutionarily newer, myelinated ventral branch enabling rapid cardiac regulation and social engagement (Porges, 2009). Together with sympathetic circuits, they form three hierarchically organized autonomic states: ventral-vagal (safety), sympathetic (mobilization), and dorsal-vagal (immobilization) (Porges, 2001). Although debated, the framework explains why some athletes remain poised while others freeze or overreact under pressure. Robust ventral-vagal tone supports calm focus and interaction, whereas dorsal dominance can manifest as collapse in extreme stress (Porges, 2009; Fisher et al., 2021). Laborde's *Vagal Tank Theory* further contributes to this understanding by viewing cardiac vagal control functioning across three systematic levels: resting, reactivity, and recovery (Laborde et al., 2018a). This model conceptualizes vagal capacity as a reservoir of self-regulatory resources, where a well-filled tank (high, resting HRV) predicts resilience and swift physiological reset after competition or stress. These theoretical frameworks provide a powerful lens through which to understand the neurophysiological underpinnings of athletic readiness and highlight the potential for targeted interventions to optimize both performance and wellbeing.

Breathing mechanics offer a direct handle on this reservoir. Slow diaphragmatic breathing at ≈ 6 breaths·min^−1^ maximizes respiratory sinus arrhythmia when heart rate accelerates during inspiration as vagal influence momentarily wanes and decelerates on expiration as vagal input rebounds (Gerritsen and Band, 2018; Malik, 1996; Vaschillo et al., 2002; Lehrer et al., 2003). Baroreceptor feedback amplifies this oscillation (Gerritsen and Band, 2018; Lehrer et al., 2003), and larger RSA amplitudes correlate with focused, relaxed states (Malik, 1996). As shown in Figure 2B, resonance-frequency breathing produces large, synchronized oscillations in heart rate and blood pressure, separated by a characteristic ≈ 5 second delay, reflecting maximal engagement of the baroreflex and vagal brake. Practices such as coherent-frequency breathing, Pranayama, and HRV biofeedback reliably elevate HRV and baroreflex sensitivity within minutes, with weeks of training producing lasting gains in resting vagal tone (Gerritsen and Band, 2018; Lehrer et al., 2003; Lehrer and Gevirtz, 2014). By contrast, rapid or shallow breathing, or prolonged inspiratory holds, suppress vagal activity and promote sympathetic arousal, underscoring breath control as a primary tool for conscious regulation of performance-critical arousal (Gerritsen and Band, 2018; Lehrer et al., 2003).

Peripheral somatic triggers can gate the brake even faster. The mammalian diving response epitomizes trigeminal-vagal synergy (Kinoshita et al., 2006). Research conducted specifically demonstrated that the full dive reflex, characterized by augmented bradycardia and sustained peripheral vasoconstriction, required the combination of face immersion and breath holding (Gooden, 1994; Kinoshita et al., 2006; Hurwitz and Furedy, 1986). As alluded with respect to the mammalian diving reflex, cold-water contact or facial submersion activates vagal and trigeminal afferents, which in turn signal brain-stem nuclei to upregulate cardiac vagal efferent activity, producing bradycardia and heightened HRV within seconds (Gooden, 1994; Ackermann et al., 2022). Healthy adults routinely show a 10%−25% heart rate drop during brief facial immersion (Ackermann et al., 2022; Schipke and Pelzer, 2001), a reflex exploited by free-divers and, anecdotally, by performers who splash their face with cold water to quell preshow jitters. Similar mechanisms underlie the oculocardiac reflex, where orbital pressure slows the heart via vagal outflow (Khurana et al., 1980; Kinoshita et al., 2006). Systematic reviews confirm that cold, apnea, and facial immersion reliably elevate vagal indices across studies (Ackermann et al., 2022). These observations highlight a broader principle in which athletes can recruit both internal (breath) and external (temperature or pressure) stimuli to engage the vagal brake quickly, providing tactical control over arousal when performance stakes are highest.

## Vagal tone, cognition, and emotional resilience

Cardiac vagal activity is a peripheral window on central self-regulation. Cross-sectional studies in healthy adults repeatedly shows that higher high-frequency HRV, an index of vagal tone, tracks superior executive performance on tasks requiring sustained attention, working memory, set-shifting, and inhibition (Hansen et al., 2003; Thayer et al., 2010; Laborde et al., 2017; Wei et al., 2024). For instance, an observational study of 143 young adults linked resting HRV with faster reaction times and fewer errors across a neuropsychological battery, suggesting that vagal control confers a broad cognitive dividend rather than a domain-specific boost (Forte and Casagrande, 2025). Neurovisceral-integration theory explains this coupling as afferent vagal fibers synapse in brain-stem nuclei that project to the pre-frontal cortex, while descending pre-frontal influences modulate vagal efferent fibers via the nucleus ambiguuus, creating a bi-directional circuit through which flexible heart rhythms mirror flexible cognition (Figure 2A) (Malik, 1996; Wei et al., 2024). Functional MRI confirms that individuals with higher HRV show stronger pre-frontal recruitment during executive challenges and tighter coupling between cerebral hemodynamics and cardiac-vagal shifts (Arakaki et al., 2023). Interventions that acutely augment vagal tone often improve cognition under stress. A single 15-min HRV-biofeedback session elevated HRV and improved network-level attention scores in highly stressed participants but not in their low-stress peers, implying a ceiling effect when baseline vagal tone is already ample (Goessl et al., 2017; Wells et al., 2012).

Beyond cognition, vagal tone underwrites emotional stability. Individuals with higher baseline HRV show muted heart rate and cortisol surges during social-evaluative stress and return to baseline more quickly, demonstrating a potent vagal brake on sympathetic arousal (Laborde et al., 2018a; Segerstrom and Nes, 2007). Six weeks of resonance-frequency HRV-biofeedback not only raises resting HRV but also reduces trait anxiety and depressive symptoms, illustrating that the parasympathetic pathway can be trained for psychological benefit (Karavidas et al., 2007). These findings align with the polyvagal view that the myelinated ventral branch fosters a felt sense of safety and social engagement; higher HRV correlates with greater interpersonal trust and empathy, both valuable in team sport settings (Porges, 2007). In contrast, low HRV is common in anxiety disorders, depression, and PTSD, and predicts vulnerability to performance choking when cognitive load and autonomic load collide (Mosley et al., 2017).

The concept of vagal flexibility refers to the autonomic nervous system's ability to rapidly withdraw parasympathetic (vagal) influence during physical or psychological challenge and to swiftly reinstate it during recovery (de Souza et al., 2020) directly examined this principle by measuring HRV during rest, exercise, and recovery phases in university professors. Their findings demonstrated that higher fitness levels, lower perceived stress, and more favorable anthropometric measures (e.g., lower waist circumference) were associated with more dynamic vagal withdrawal and rebound hallmarks of vagal flexibility. This flexibility not only reflects a resilient autonomic response but is also crucial for cardiovascular safety, reducing the window of exposure to arrhythmogenic risk post-exercise (de Souza et al., 2020). Complementing this, Langdeau emphasized that efficient sympatho-vagal balance plays a vital role in physiological responsiveness and recovery, especially in trained athletes (Langdeau et al., 2000). Together, these studies underscore that vagal flexibility is not merely a theoretical construct but a measurable and trainable physiological trait that integrates stress, fitness, and autonomic control into a single index of adaptive health.

Vagal regulation, particularly as indexed by cardiac vagal tone and HRV, extends beyond individual self-regulation to play a critical role in shaping social engagement and group dynamics. According to the polyvagal theory, higher baseline vagal tone supports adaptive self-regulation strategies and promotes prosocial behaviors such as seeking social support and emotional cooperation (Geisler et al., 2013). These findings align with evidence that vagal-mediated HRV facilitates not only the regulation of distress but also the capacity for meaningful social interaction, making it foundational to social bonding and emotional resilience. At the group level, McCraty (2017) introduced the concept of *social coherence*, where the physiological synchronization of HRV among group members corresponds with improved collective functioning, communication, and emotional alignment (McCraty, 2017). In both laboratory and naturalistic settings, HRV synchrony has been associated with increased cooperation, compassion, and trust among group members, even in the absence of verbal interaction. Moreover, physiological entrainment, such as synchronized heart rhythms, has been observed between parents and infants, classmates, musicians, and even spectators and performers during emotionally intense shared experiences (McCraty, 2017). These phenomena suggest that vagal flexibility is not only an individual marker of health but also a dynamic mechanism through which social organisms coordinate behavior, share emotional states, and build cohesive communities. Taken together, these findings position vagal tone and flexibility as foundational substrates for both the *thinking game* and the *emotional game*. Athletes who cultivate a robust, adaptable parasympathetic system gain sharper executive control, steadier emotions, and stronger interpersonal alignment, all prerequisites for consistent high performance.

## Regulation of psychophysiological arousal for functional performance

Athletic performance demands a finely tuned balance between sympathetic drive and parasympathetic restraint. More than a century ago, Yerkes and Dodson (1908) framed this trade-off as an inverted-U performance function with arousal or stress building to improve performance until a tipping point is reached, after which excess performance pressure degrades precision and judgment (Figure 1A). Modern autonomic science locates the fulcrum of that curve in the vagus nerve. Resting cardiac-vagal tone establishes an athlete's baseline arousal; a strong vagal brake keeps resting heart rate low and cortical networks calm, creating physiological headroom to upregulate during competition (Laborde et al., 2018a; Malik, 1996). By contrast, chronically low HRV leaves the baseline already elevated, so even modest sympathetic surges propel the performer onto the descending limb of the curve where tremor, narrowed attention, and cognitive rigidity appear (Gullett et al., 2023).

Controlled laboratory research supports this mechanistic link. When individuals with high resting HRV undertake a stressor such as timed mental arithmetic, heart rate and catecholamine rises remain proportionally smaller and executive accuracy is preserved; low-HRV counterparts show steeper physiological slopes and more errors (Hansen et al., 2003; Goessl et al., 2017). Field studies extend the pattern showing elite rifle shooters who sustain HRV within 5% of baseline during the pre-shot routine display superior hit rates, whereas those whose vagal tone collapses under pressure show clutch-to-choke reversals (Ortega and Wang, 2017). Similar effects have been demonstrated in precision motor tasks under high-pressure conditions, where individuals who exhibited greater vagal withdrawal from baseline to task, indexed by cardiac vagal reactivity, achieved higher dart scores and made fewer errors during concurrent cognitive tasks, suggesting flexible vagal modulation supports both motor precision and executive control (Mosley et al., 2017). Suggesting that vagal flexibility, the ability to release the brake briskly for action and re-engage it during pauses, is an overlooked pillar of skill execution and mastery.

HRV monitoring has therefore become a surrogate gauge of the real-time arousal landscape. Daily waking HRV provides a readiness score in which deviations below an individual's rolling average warn of sympathetic overload, infection, or sleep debt (Schipke and Pelzer, 2001; Plews et al., 2013). Coaches increasingly integrate HRV readings into daily training prescriptions to optimize autonomic adaptation and performance gains. When morning HRV is suppressed, falling below a personalized rolling baseline, athletes are assigned low-intensity sessions or rest to support recovery. Conversely, when HRV rebounds or remains within the athlete's smallest worthwhile change, high-intensity training can be performed safely. This adaptive model has proven more effective than standardized training protocols. For example, HRV-guided runners in both short- and long-term interventions showed greater improvements in maximal running velocity, endurance performance, and aerobic capacity, despite often performing fewer intense sessions (Kiviniemi et al., 2007; Vesterinen et al., 2016). These findings support HRV-based training as a responsive and individualized method for managing load, reducing non-responder rates, and enhancing cardiorespiratory fitness.

Importantly, more vagal tone is not always better. While low HRV is consistently linked to poor health outcomes, stress, and overtraining (Bellenger et al., 2016), unusually high HRV is not necessarily optimal either. In some endurance athletes, a state of parasympathetic overreaching has been observed, marked by elevated resting vagal activity, persistent fatigue, and diminished performance, suggesting maladaptive recovery rather than enhanced fitness (Meeusen et al., 2013; Le Meur et al., 2013). Additionally, shifts in HRV patterns have been associated with autonomic nervous system dysfunction, which can impair cardiovascular regulation and training responsiveness (Bellenger et al., 2016). While HRV remains a valuable tool for monitoring readiness and adaptation, its interpretation should consider the broader physiological and psychological context rather than assuming that more is always better.

Rapid self-regulation strategies can help performers return to an optimal physiological state during competition. Engaging in slow diaphragmatic breathing particularly at a pace of six breaths per minute with extended exhalation, has been shown to enhance vagal activity and reduce systolic blood pressure within minutes (Van Diest et al., 2014; Afify, 2023). Cold-water facial immerse on activates the trigeminal–vagal diving reflex, resulting in a transient bradycardic response and shift toward parasympathetic dominance, with heart rate reductions of up to 15% observed in some cases (Kinoshita et al., 2006). Mental reframing, shifting one's internal narrative from perceiving a situation as a threat to viewing it as a challenge, can also influence autonomic regulation. According to the polyvagal perspective, such cognitive strategies recruit higher cortical circuits that preserve or restore cardiac vagal tone via the social engagement system, helping stabilize physiological arousal in high-pressure moments (Porges, 2007). Together, these techniques offer athletes practical tools to modulate vagal state mid-competition, reinforcing the idea that autonomic control is not fixed but trainable and responsive to intentional input.

Long-term autonomic training targets both tonic vagal tone and phasic flexibility. Regular practice of resonance frequency breathing, typically 10 min daily over a period of several weeks, has been shown to significantly increase resting HRV and enhance baroreflex sensitivity, a marker of improved autonomic regulation (Lehrer et al., 2003; Shaffer and Meehan, 2020). While these exercises focus on elevating baseline parasympathetic tone, complementary strategies such as short breath holds or brief maximal sprints followed by mindful recovery are used to deliberately train vagal withdrawal. These methods expand the autonomic response range, preparing the performer to both engage and recover more efficiently. Contemporary HRV-biofeedback platforms increasingly embed these principles, providing structured feedback to reinforce both sustained HRV elevation and rapid modulation capacity.

Taken together, the modern understanding of arousal regulation is no longer defined by a static curve but by a dynamic, adaptable landscape shaped by vagal tone, autonomic flexibility, and situational context. Continuous monitoring tools make this internal terrain visible, while interventions such as resonance breathing, cold exposure, cognitive reappraisal, and HRV biofeedback give athletes the means to navigate it in real time. Anchoring training and recovery decisions to individualized autonomic data allows practitioners to keep performers balanced near the apex of the inverted-U, alert but not anxious, composed yet primed, where physical precision and cognitive clarity optimally converge (Figure 1).

## Vagus nerve stimulation: from clinical neuromodulation to applied ergogenics

For decades, invasive VNS has been an accepted therapy for drug-resistant epilepsy and major depression, achieved by surgically wrapping an electrode around the cervical vagus and delivering intermittent pulses (Handforth et al., 1998). Incidental reports of brighter mood, sharper attention, and improved autonomic balance in these patients sparked the question: Can stimulating vagal afferents in healthy people enhance cognition, recovery, and ultimately, sport performance? That question now drives a rapidly expanding literature centered on non-invasive methods of tVNS, which excites the same brainstem nuclei through the skin, either at the external ear via the auricular branch of the vagus nerve (ABVN) or at the neck via cervical branches of the vagus (Urbin et al., 2021; Sharon et al., 2020; Frangos et al., 2015; Kraus et al., 2007; Tyler et al., 2015, 2019; Croft et al., 2025). These approaches are like conventional transcutaneous electrical nerve stimulation (TENS), but use smaller, custom electrodes to deliver low-intensity pulsed currents to cranial nerve targets on the head, neck, face, and ear. The evolution from invasive to non-invasive stimulation is now reflected in wearable technologies that access cranial nerve afferents externally using these modified TENS approaches to achieve desired outcomes as described below.

**Figure 3:**
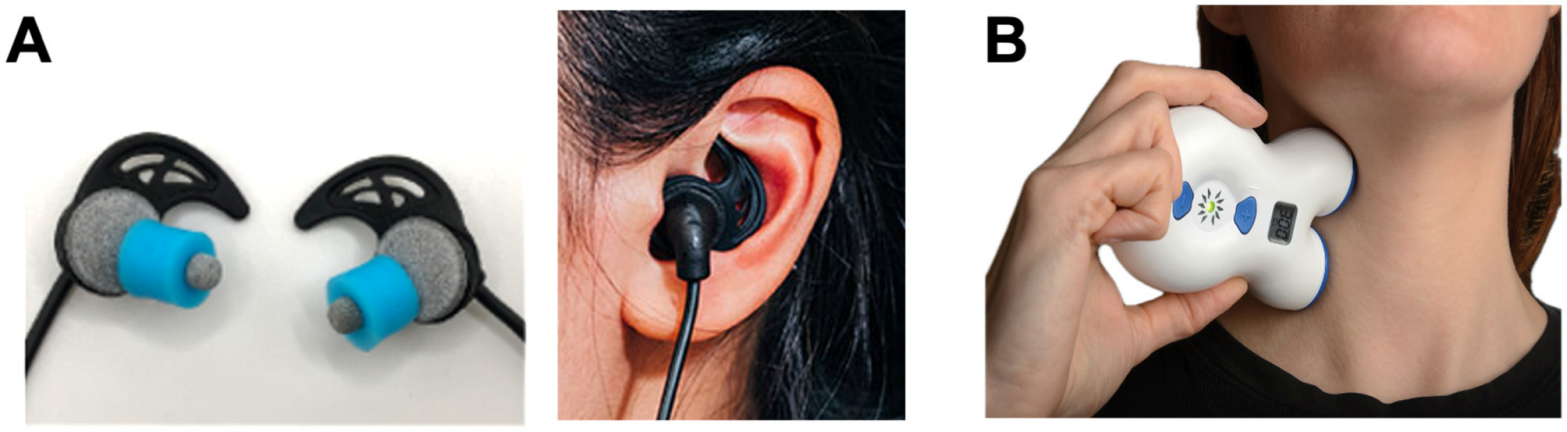
Transcutaneous electrical nerve stimulation methods of non-invasive vagal modulation. Modern non-invasive vagus nerve stimulation methods enable the targeting of vagal fibers through the skin using compact, user-friendly embodiments of transcutaneous electrical nerve stimulation (TENS) devices designs. These TENS-like devices deliver low-intensity (< 10 mA) pulsed electrical currents through the skin to safely modulate vagal activity. **(A)** Shown are photographs of a transcutaneous auricular vagus nerve stimulation (taVNS) device (BRAIN Buds; IST, LLC) that utilizes conductive hydrogel earbud electrodes to access the auricular branch of the vagus lining the acoustic meatus of the external ear enabling comfortable and precise self-administration (Tyler, 2025). **(B)** A transcutaneous cervical vagus nerve stimulation (tcVNS) device (GammaCore; ElectroCore, Inc.) is shown applied to the neck to stimulate the cervical branch of the vagus nerve using metal contact electrodes (Silberstein et al., 2016). Together, these examples illustrate the shift toward modular, portable neuromodulation tools for clinical and human performance applications.

Acting on noradrenergic pathways to subdue sympathetic reactivity, transcutaneous trigeminal and vagal stimulation at tens of kHz has been shown to reduce salivary alpha amylase (a biomarker of NE activity), suppress galvanic skin conductance, increase skin temperature via sympathetic sudomotor relaxation (vasodilation), and decrease subjective stress in response to an electrical shock-mediated fear conditioning paradigm in healthy humans (Tyler et al., 2015). Transcutaneous auricular vagus nerve stimulation (taVNS) devices use different types of surface electrodes ranging from electrode ear-clips to earbud-style electrodes that target ABVN fibers via the external acoustic meatus, tragus, or cymba conchae of the external ear, providing self-directed neuromodulation in a comfortable, modular format (Tyler, 2025) (Figure 3A). Transcutaneous cervical vagus nerve stimulation (tcVNS) devices, first developed as an FDA-cleared treatment for headache, are applied to the side of the neck to stimulate the cervical vagal branches (Figure 3B). Both taVNS and tcVNS have been demonstrated to treat a wide range of clinical indications spanning: mood disorders like depression, anxiety and post-traumatic stress disorder; movement disorders like essential tremor and Parkinson's disease; neurophysical conditions and injuries like traumatic brain injury, spinal cord injury, and stroke; inflammatory conditions including pain and several autoimmune disorders; and other neurologic and neuropsychiatric disorders (Butt et al., 2019; Croft et al., 2025; Kelly et al., 2022; Kim et al., 2022; Yap et al., 2020; Yuan and Silberstein, 2015). More globally, however, these non-invasive neurotechnologies represent a significant shift toward scalable, user-friendly VNS methods suitable for not only clinical populations, but also for healthy individuals aiming to improve performance, recovery, and general wellness (Tyler, 2025). The specific stimulation sites targeted at the external ear (taVNS) and the side of the neck (tcVNS) provide non-invasive access to branches of the vagus nerve that contain afferent fibers projecting to the ascending reticular activating system (RAS) including the nucleus of the solitary tract (NTS) and LC in the brainstem. These approaches enable direct neuromodulation conduits to core, deep-brain functions without surgical intervention. Transcutaneous VNS elicits a reproducible cascade where afferent fibers synapse in the brainstem nuclei of the RAS including the NTS and LC eliciting the release of norepinephrine (NE) and acetylcholine that act to adjust cortical gain by sharpening neural signal-to-noise ratios while concurrently activating efferent vagal pathways that clamp sympathetic arousal, slow the heart, and reduce inflammation (Figure 2A).

Transcutaneous VNS has recently gained attention for its safely modulate autonomic nervous system activity, inflammation, neuroplasticity, attention, stress, learning, mood, and sleep, by biasing the activity of brain nuclei and neurotransmitters known to regulate these processes, such as the LC and NE, respectively (Urbin et al., 2021; Tyler, 2025; Kim et al., 2022; Liu et al., 2020; Phillips et al., 2021; Olsen et al., 2023). Many investigations over the last decade have shown that both tcVNS and taVNS can reduce the sympathetic nervous system activity, as well as the psychological and neurophysiological symptoms of stress (Szeska et al., 2025; Trifilio et al., 2023; Bretherton et al., 2019; Machetanz et al., 2021a,b; Gurel et al., 2020; Moazzami et al., 2023; Sommer et al., 2023). These controlled studies have clearly demonstrated that both taVNS and tcVNS can produce significant changes in bottom-up neurophysiological arousal, leading to improved cognitive control and impulse control during emotional tasks, which may translate to enhanced athletic performance under intense competitive pressure. The ability of taVNS to dampen stress responses underlies its ability to improve performance under high cognitive and emotional loads. For example, taVNS has been shown to improve action control performance and response selection when task demands are high (Jongkees et al., 2018). Collectively, these data suggest taVNS provides an approach to tune LC/NE activity across different states of arousal for optimizing performance depending on several variables (Figure 1B).

Development of high levels of sport-specific executive functioning, including skill learning and memory, task-based attention, and rapid decision making are critical for elite athletes to achieve high levels of performance. Several studies have shown that transcutaneous VNS can enhance learning and memory based on to its ability to modulate human cortical arousal, hippocampal function, and attention (Jacobs et al., 2015; Sharon et al., 2020; Tyler et al., 2019; Trifilio et al., 2023; Chen et al., 2023; Rufener et al., 2018; Miyatsu et al., 2024). For instance, recent taVNS studies show it can significantly improve motor action planning, enhance motor sequence learning, and improve associated motor cortex efficiency (Chen et al., 2024, 2022). It has also been demonstrated that taVNS can improve human working memory (Sun et al., 2021) and cognitive flexibility (Borges et al., 2020) both critical skills necessary for real-time athletic performance. Athletes may leverage these mechanisms and the ability of transcutaneous VNS to sustain clarity, enhance cognitive function, and maintain emotional control under conditions of highly competitive pressure, fatigue, or distraction (Figure 1).

To enhance learning, reduce stress, or improve sleep, it is critical that tVNS interventions not overstimulate LC/NE activity or produce off-target effects that can arise from excessive or uncomfortable stimulus sensations, thereby overtaking intended taVNS performance outcomes (Tyler et al., 2019; Tyler, 2025; Miyatsu et al., 2024; Jigo et al., 2024). Restated, transcutaneous VNS can both activate and suppress sympathetic activity (stress) depending on many variables, including the electrode interface, user sensation and comfort, stimulus frequency, pulse duration, ease of use, the user's baseline arousal, and other variables (Figure 1B). This has been observed in studies evaluating the influence of cognitive load and transcutaneous VNS on task-based performance, brain wave activity, HR/HRV, and pupillometry as measures of neurophysiological arousal (Urbin et al., 2021; Sharon et al., 2020; Tyler et al., 2019; Phillips et al., 2021; Faller et al., 2019; Pandža et al., 2020). Most studies to date implementing transcutaneous VNS for clinical applications implement stimulus frequencies ranging from 10–30 Hz using methods that produce suprathreshold sensory effects. Increased feeling of the electrical stimulus tends to increase sympathetic activity, whereas just noticeable or subperceptual stimulation tends to decrease sympathetic arousal. It has been found that taVNS can produce differential effects on pupil diameter, which is a known biomarker that reflects LC/NE activity and task-based performance in an inverted-U shape manner (Faller et al., 2019), HRV, and cortical arousal across a range of stimulus frequencies (10–3,000 Hz) and amplitudes (0–15 mA) (Urbin et al., 2021; Sharon et al., 2020; Tyler et al., 2019; Pandža et al., 2020).

Interestingly, kHz high-frequency stimulation (1–20 kHz) can reduce stimulus sensations while enabling higher peak currents to be delivered to cranial nerves in a manner that remains capable of reducing sympathetic activity and triggering widespread changes in cortical activity (Tyler et al., 2015, 2019; Mao et al., 2022). Recent evidence demonstrates that a single session of sub-perceptual taVNS at 20 kHz for 15 min can produce significant changes in the functional connectivity of the prefrontal cortex, cingulate cortex, and insula (Mao et al., 2022) regions involved in regulating emotional reactivity and cognitive control. Other studies have shown that 300 Hz taVNS can produce non-linear effects on pupil diameter across a range of stimulus intensities, including when subthreshold sensory effects were produced (Urbin et al., 2021; Phillips et al., 2025). These observations indicate that future transcutaneous VNS efforts aimed at developing human performance enhancers should focus on optimizing the neurostimulation variables and parameters required to optimize the stimulus sensations evoked, user comfort, ease of use, ecological validity, and situational efficacy (Tyler, 2025; Tyler et al., 2025).

Other outcomes attributed to taVNS are useful for enhancing athletic performance and recovery. The cholinergic anti-inflammatory pathway (CAIP) involves the signaling of cytokine activity by visceral vagal afferents, which activates homeostatic brain regions and in turn the spleen via cholinergic vagal efferent fibers that act to reduce pro-inflammatory cytokine production. Several lines of evidence demonstrate that electrical VNS, including taVNS and tcVNS, can reduce inflammation by acting on the CAIP (Czura and Tracey, 2005; Pavlov and Tracey, 2012; Kelly et al., 2022; Liu et al., 2020). In a randomized crossover trial, seven consecutive days of bilateral taVNS (30 min/day, 25 Hz) led to a 3.8% increase in VO_2_ peak and a 6-watt gain in peak work rate compared to sham stimulation, alongside reductions in pro-inflammatory markers such as IL-1β (Ackland et al., 2025). Importantly, post-exercise blood sampled from the stimulation arm showed a muted pro-inflammatory cytokine response, aligning with activation of the CAIP and suggesting an accelerated recovery milieu (Ackland et al., 2025).

Another recent study examined the influence of unilateral and bilateral taVNS on performance, pain, fatigue, and lactic acid levels in response to four consecutive days of 30 min of maximal exertion stationary cycling in healthy, young adults (Hatik et al., 2023). Hatik et al. (2023) found that taVNS after exercise can decrease fatigue, pain, and lactic acid levels, while increasing parasympathetic activity without producing undesirable effects on pulse and blood pressure. Studies into the effects of 100 Hz taVNS on hemodynamics and autonomic nervous system function during exercise stress tests have shown reduced HR at maximal exercise and 1 min following maximal exertion (Yoshida et al., 2025). Yoshida et al. (2025) also found taVNS significantly increased the stroke volume and decreased total peripheral resistance at maximum exercise. Furthermore, taVNS produced a decrease in the LF/HF HRV ratio, reflecting reduced sympathetic dominance at rest and at maximum exercise (Yoshida et al., 2025). Collectively, these observations regarding the influence of taVNS lend credence to hypotheses that the strength of cardiac vagal activity is a causal determinant in our ability to exercise (Gourine and Ackland, 2019; Laborde et al., 2018b).

It has been hypothesized that physical exertion is limited by a central governor in the brain that receives afferent inputs from physiological systems, and that the conscious awareness of this activity is the major contributor to fatigue and failure of skeletal muscle (Noakes et al., 2005). These neural mechanisms prevent higher levels of exertion, although they are physically possible. This mental block is believed to be an evolutionary safety feature that prevents injury during intense physical activity. An interesting possibility is that taVNS can help athletes overcome their central governor to achieve higher levels of performance. It is in fact believed that one may overcome perceived physical limitations imposed by a central governor through psychological operations, such as those underlying the principles of positive psychology and mind over body practices. In fact, many endurance athletes, extreme athletes, and sport professionals are recognized for their ability to achieve feats that are seemingly impossible and beyond their physical limitations. These moments are often marked by athletes when they enter a *flow state* (Csikszentmihalyi, 1988, 1990). In positive psychology, the attainment of flow is marked by a period of highly positive productivity and peak performance that feels effortless and enjoyable to high-functioning individuals and elite athletes (Csikszentmihalyi, 1990; Harris et al., 2021). Investigations have revealed that the ability to attain flow varies as an inverted-U shape function across levels of stress and sympathetic arousal (Peifer et al., 2014). By contrast, parasympathetic activation is linearly and positively related to the ability to achieve flow, indicating that modulation of both branches of the autonomic nervous system facilitates the flow experience (Peifer et al., 2014). Furthermore, several lines of evidence show that LC/NE activity is a key neurophysiological variable gating the flow experience (van der Linden et al., 2021). This involvement of LC/NE activity is mechanistically consistent with observations demonstrating taVNS can enhance flow states (Colzato et al., 2017).

Considering the sum of evidence described, transcutaneous VNS may represent an *ultima thule* for helping individuals overcome neurophysiological and psychological barriers to achieving peak performance. More rigorous studies are required to advance transcutaneous VNS, particularly for elite athletes. These studies need to evaluate both acute and long-term outcomes across physical, physiological, and psychological variables while standardizing methods of transcutaneous VNS intended to enhance athletic performance and recovery. Currently, from a practical standpoint, the scientific understanding of transcutaneous VNS suggests it is a promising application used alone or as a complementary tool for improving athlete performance and mental health.

## Autonomic training strategies: controlled breathing, HRV biofeedback, and environmental exposure

**Figure 4:**
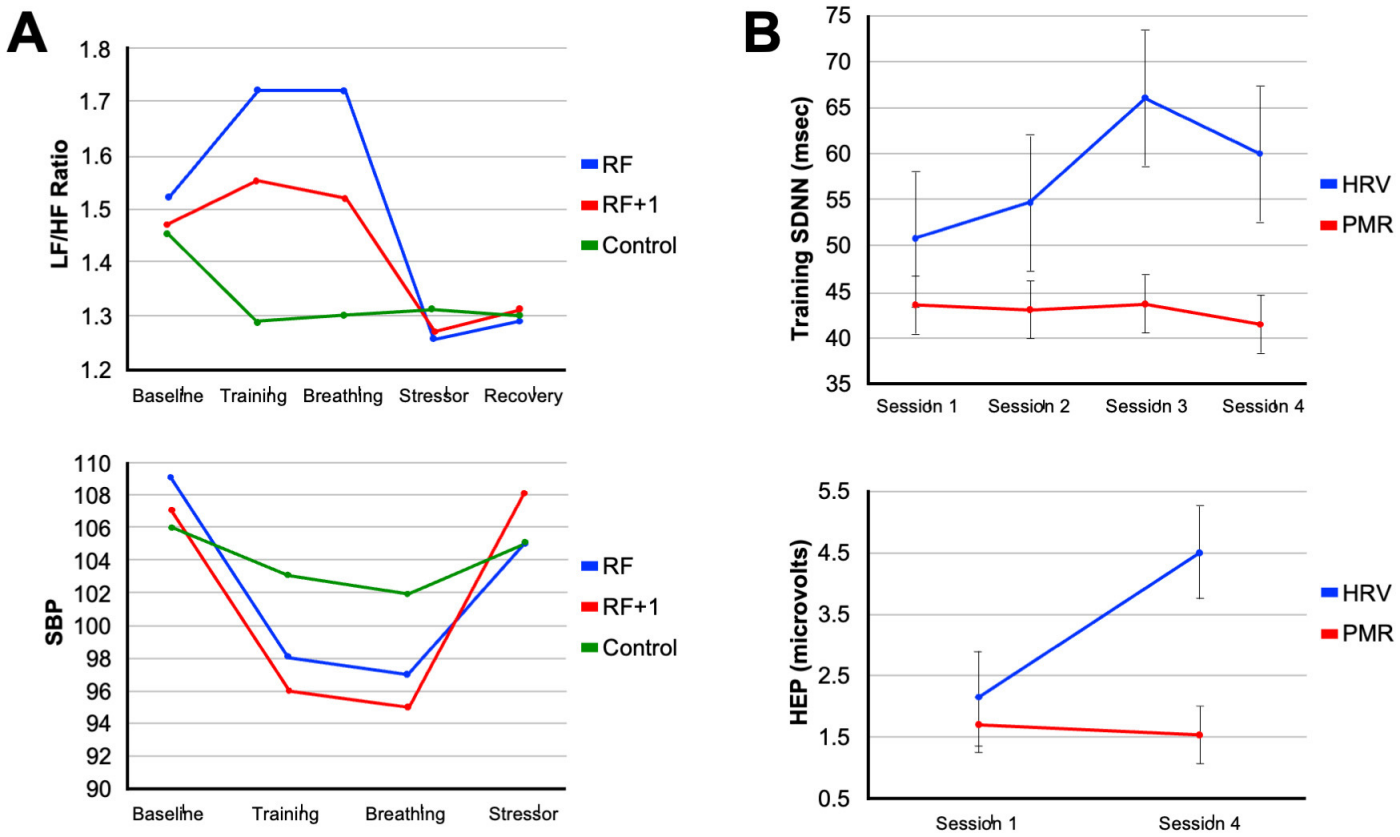
Modulation of autonomic arousal by heart rate variability biofeedback training. **(A)** The line plots illustrate the specificity of resonance frequency breathing (RF) on autonomic regulation. The *top panel* shows that participants in the RF group exhibited the greatest increase in LF/HF ratio during the breathing phase, indicative of optimized baroreflex resonance, while the RF+1 and control groups showed minimal or negative changes. The *bottom panel* shows systolic blood pressure (SBP) trends, where both RF and RF+1 groups exhibited reductions during training, but only the RF group maintained attenuated blood pressure reactivity during a subsequent stressor (Steffen et al., 2017). **(B)** The line plots illustrate the cumulative neurophysiological effects of HRVBFT across training sessions. The *top panel* shows increases in HRV amplitude over four sessions in the HRVBFT group (HRV), with little change in the progressive muscle relaxation (PMR) control group. The *bottom panel* shows significant increases in heartbeat-evoked potential (HEP) amplitude, an EEG marker of interoceptive engagement, only in the HRVBFT group, suggesting enhanced brain-body integration and vagal afferent signaling following HRVBFT (Lehrer and Gevirtz, 2014). **(A)** Were adapted from Steffen et al. (2017) and (**B**) from Lehrer and Gevirtz (2014).

The ability of an elite athlete to self-regulate and control arousal is one of the most critical psychological factors influencing their ability to achieve peak performance (Anderson et al., 2014). Among the spectrum of non-electrical approaches to vagal conditioning, slow-paced breathing remains the most direct lever. Each inhalation suppresses cardiac vagal outflow while each exhalation reinstates it (Figure 2B). Exaggerating this rhythm by respirating at roughly six breaths per minute maximizes respiratory sinus arrhythmia and baroreflex gain, the twin signatures of a responsive parasympathetic system (Gerritsen and Band, 2018). Laboratory studies indicate that a single 15-min session of individually determined resonance frequency breathing can increase the LF/HF ratio of HRV and decrease blood pressure response during a cognitive stressor (Steffen et al., 2017). These effects can be clearly observed in Figure 4A, which shows that participants in a resonance frequency breathing group (RF) exhibited increases in LF/HF ratio during the breathing phase, while phase shifted respiration (RF+1) and control groups showed little to no HRV changes. Notably, systolic blood pressure (SBP) also declined most during breathing in the RF group and remained more stable during a subsequent stressor, indicating attenuated cardiovascular reactivity, a hallmark of *vagal resilience* (Figure 4A) (Steffen et al., 2017).

Joining rhythmic breathing practices with taVNS has gained attention recently. Some approaches known as respiratory gated auricular VNS (RAVNS) have been shown to differentially alter vagal engagement and brain activity depending on the phase (inhalation vs. exhalation) of the respiratory cycle when stimulation is delivered (Garcia et al., 2022; Szulczewski et al., 2023; Szulczewski, 2022; Garcia et al., 2021; Sclocco et al., 2019). It remains undetermined whether RAVNS procedures make any difference in the magnitude of potential performance benefits compared to conventional taVNS methods. Respiration has a clear and natural, modulatory effect on cardiac vagal activity (Dergacheva et al., 2010), but whether external VNS can differentially enhance these effects for significantly improving performance remains to be determined through systematic studies that carefully vary stimulus approaches and parameters as discussed above. Integrated training paradigms combining taVNS with focused breathwork and contemplation is certainly an enticing triad of performance optimization approaches for enhancing self-regulation and cardiac vagal activity. A single session of slow-paced breathing has been shown to improve executive function in young adults, although this effect was not found to be mediated by changes in RMSSD (Laborde et al., 2018a). Incorporating resonance breathing into daily practice over several weeks can also lead to improvements in resting HRV and cognitive function, suggesting the potential for sustained autonomic changes with repeated practice (Lehrer and Gevirtz, 2014; Chaitanya et al., 2022). The on-demand nature of breathwork and its performance implications are straightforward. Athletes and coaches can use short shallow breaths to engage sympathetic activity while implementing slow rhythmic and deep diaphragmatic to stimulate parasympathetic dominance under different situations. They can remain confident that the maneuvers can modulate vagal capacity for the upcoming cognitive or metabolic demands.

Heart rate variability biofeedback training (HRVBFT) is another intervention that teaches individuals to regulate their breathing and HR to increase HRV, thereby enhancing vagal tone and promoting autonomic flexibility (Lehrer and Gevirtz, 2014). HRVBFT is a well-known method of enhancing cardiac vagal activity to enhance sports performance (Jiménez Morgan and Molina Mora, 2017). Meta-analyses indicate that HRVBFT is associated with a large reduction in self-reported stress and anxiety across various populations (Goessl et al., 2017). Studies in college students and graduate students have shown that computer-based HRVBFT programs can lead to significant decreases in anxiety and negative mood (Henriques et al., 2011; Lee et al., 2015). The mechanism is thought to involve the training of autonomic reflexes and the restoration of autonomic homeostasis, which supports emotional regulation and can improve responses to stress (Lehrer and Gevirtz, 2014; Goessl et al., 2017).

Training-related changes in autonomic function are evident in studies comparing HRVBFT to active control interventions. For example, repeated sessions of HRVBFT produce progressive increases in HRV amplitude over time, while control participants engaging in progressive muscle relaxation (PMR) techniques show minimal change (Lehrer and Gevirtz, 2014). Additionally, only the HRVBFT group demonstrates increases in heartbeat-evoked potential (HEP) amplitude, an EEG-based marker of interoceptive awareness and vagal afferent engagement, suggesting that HRVBFT strengthens both autonomic output and brain-body signaling (Lehrer and Gevirtz, 2014) (Figure 4B). Corroborating these outcomes, taVNS has been shown to enhance cardiac awareness and interoception (Zhao et al., 2022; Paciorek and Skora, 2020; Villani et al., 2019). These findings indicate HRVBFT and taVNS share common mechanisms as stress-reduction approaches and neuromodulatory training methods. Evidence demonstrates that these easy-to-implement methods can improve cardiac vagal activity, cognitive-emotional control, and self-regulation through enhanced central processing of internal physiological cues. While still in its early stages for elite athletes, initial evidence from a peer-reviewed case study in a young competitive golfer demonstrated that HRV biofeedback training over 10 weeks resulted in increased HRV, decreased anxiety and negative mood states, and a 15-stroke improvement in golf performance, despite no changes to physical training, highlighting its potential as a tool to manage competitive stress and enhance outcomes (Gamaiunova et al., 2019). Golf is widely recognized as a mental game requiring a relaxed state of mind and calm neurophysiological arousal. Interestingly, the first use of taVNS in professional sports was to reduce performance anxiety for an athlete competing in a professional golf putting competition (Lagos et al., 2008). These approaches are proving useful for athletes whose ability to auto-regulate physiological and emotional responses is crucial for managing competitive stress and optimizing performance (Anderson et al., 2014).

Environmental stimuli also represent a category of interventions that can influence autonomic function and vagal tone. The mammalian diving reflex, for instance, is a physiological response triggered by stimuli such as cold-water exposure, particularly during full facial submersion. This response involves a coordinated set of changes, including bradycardia (slowing of HR), peripheral vasoconstriction, and a shift in autonomic balance toward increased parasympathetic activity, mediated by the trigeminal (CN V) and vagus nerve (CN X). A systematic review and meta-analysis confirms that the diving response elicits significantly increased cardiac vagal activity, as measured by HRV root mean square of successive differences (RMSSD), producing moderate to large positive effect sizes during exposure compared to resting conditions (Ackermann et al., 2022). The meta-analysis included studies examining triggers such as face immersion or cooling, SCUBA diving, and total body immersion, and found that total body immersion had a significantly larger effect on RMSSD than simply cooling the forehead (Ackermann et al., 2022). In another study, we investigated the influence of breathwork, breath-hold diving, and full-body underwater physical activity on performance and mental health variables across a range of athletes and sports in individuals with experience ranging from recreational to professional. An additional psychological aspect of the Deep End Fitness (DEF) training program we studied included fear or stress inoculation due to hunger for air associated with underwater, breath-hold workouts (Cansler et al., 2023). Stress and fear inoculation methods are useful for teaching individuals and teams to remain calm under intense performance pressure. Following 4 to 6 weeks of DEF training, athletes had significant reductions in stress and anxiety with significant improvements in positive coping compared to controls, which led to personal and athletic performance enhancements (Cansler et al., 2023). We propose that teaching breathwork and stress coping skills utilized in the face of fear (hunger for air) during physical activity performed while breath hold diving reinforces auto-regulation skills involving cardiac vagal mechanisms stimulated by the mammalian diving reflex. Understanding how specific environmental stimuli and contexts modulate the autonomic nervous system offers another avenue for exploring non-pharmacological methods to influence physiological and psychological states for enhancing performance, stress management, and emotional regulation.

**Figure 5:**
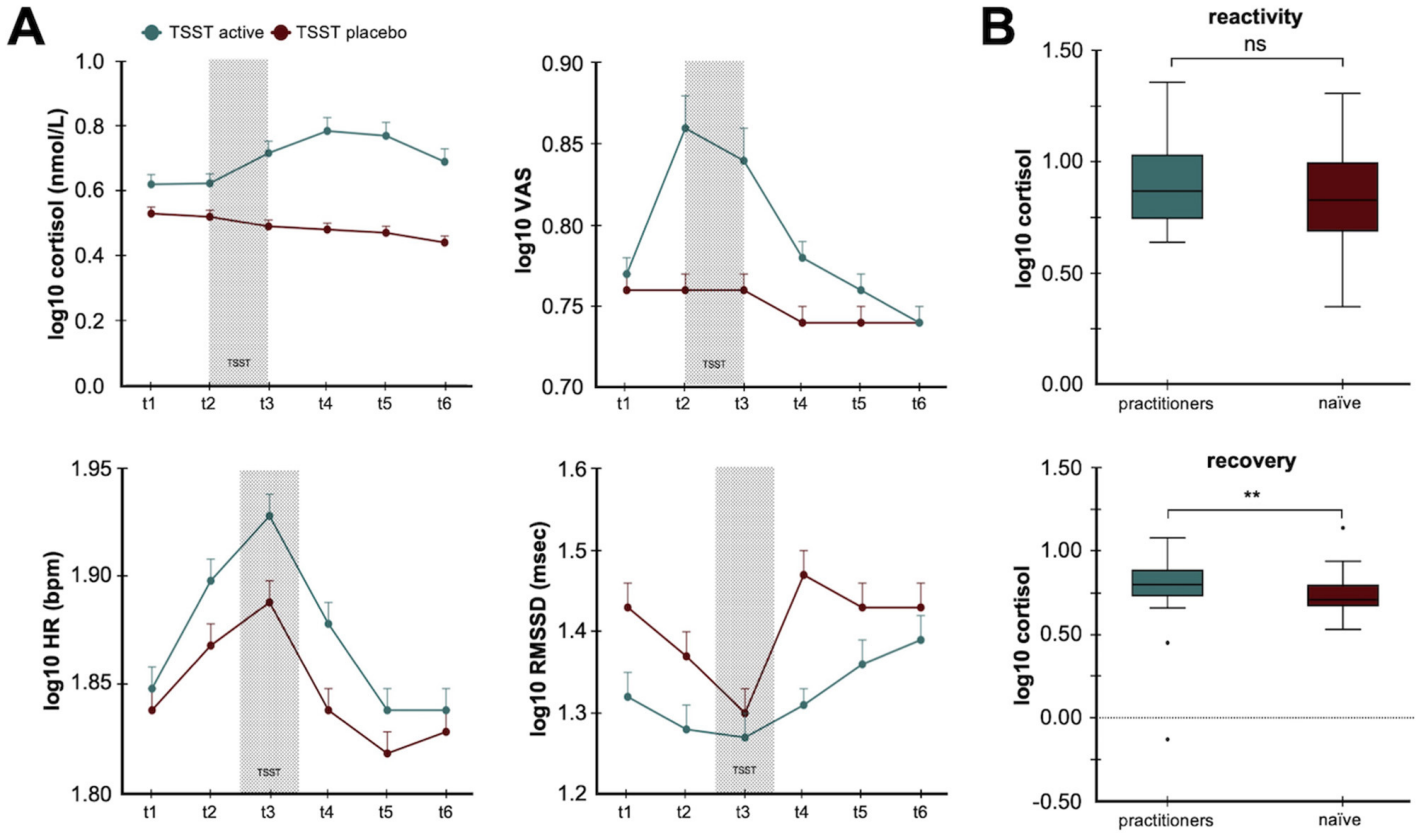
Contemplative practice reduces psychophysiological responses to stress. **(A)** The line plots illustrate how long-term contemplative practice modulates both the hypothalamic pituitary axis (HPA) and autonomic responses to psychosocial stress. The *top panels* show cortisol levels and subjective stress ratings while the *bottom panels* illustrate heart rate (HR) and heart rate variability root mean square of successive differences (RMSSD) in response to active and placebo Trier Social Stress Tests (TSST). **(B)** The histograms illustrate differences in cortisol concentrations for experienced meditation practitioners compared to naïve controls in response to stress (*top*) and during the recovery period (*bottom*). The data show acceptance-based coping strategies from meditation experience result in faster cortisol recovery (Taublieb, 2018). The figures are adapted from Gamaiunova et al. (2019). ^**^p < 0.05.

Contemplative practices and meditation are other natural, autonomic training strategies that have been investigated for their impact on neurophysiological markers and psychological states. Intensive mindfulness meditation training, such as Vipassana, has been associated with improvements in self-reported wellbeing and reductions in measures like depression and stress, alongside complex changes in HRV that may reflect altered autonomic function during meditation practice (Krygier et al., 2013). Experimental research has also shown that experienced meditators exhibit attenuated autonomic and endocrine responses to acute stressors. For example, when exposed to the Trier Social Stress Test (TSST), long-term meditation practitioners show lower cortisol and heart rate responses, smaller reductions in HRV, and lower subjective stress ratings compared to naïve, non-meditators (Figure 5) (Taublieb, 2018). Importantly, higher levels of acceptance of a trait often cultivated through meditation, predict faster cortisol recovery following the stressor (Taublieb, 2018) (Figure 5). This suggests that meditation can shape both physiological reactivity and recovery through top-down modulation of stress-related systems, including vagal tone and the HPA axis.

Different types of meditation may have distinct effects on cardiac activity. For example, studies comparing breathing meditation, loving-kindness meditation, and observing-thoughts meditation have shown variations in heart rate and HF-HRV responses, suggesting that not all meditation practices elicit the same physiological state and that effects can change with training over time (Lumma et al., 2015). Furthermore, randomized controlled trials comparing mindfulness meditation to other stress-reducing interventions like physical activity and HRV biofeedback have found that mindfulness meditation can be equally effective in reducing perceived stress, anxiety, and depressive symptoms, as well as improving psychological wellbeing, highlighting its potential as a self-help intervention for stress complaints in various populations (van der Zwan et al., 2015). A unifying theory detailing these variables is the *Respiratory Vagal Stimulation Model of Contemplative Activity*, which accounts for variables underlying both acute and long-term, vagally mediated changes in autonomic balance and function when controlled, rhythmic breathing is combined with meditative practices (Gerritsen and Band, 2018). The literature and performance models clearly suggest that meditation and controlled breathing influence the autonomic nervous system by reducing its impact on performance stress and anxiety, thereby representing a useful set of tools for athletes who face significant mental and physiological demands.

Autonomic training strategies such as breath control, HRV biofeedback, and meditation, along with targeted environmental exposures like those involved in the diving response, offer evidence-backed, non-pharmacological means of enhancing vagal capacity. These approaches can contribute to improved physiological and psychological regulation, underpinning composure, focus, and recovery. Rather than replacing fundamental physical preparation, these methods serve to create autonomic headroom, potentially allowing training adaptations to occur with fewer setbacks. They allow for mental preparation while being able to support performance by enhancing mastery during skills training and competition. Collectively, these strategies provide compelling evidence that modulation of individual performance and physiological responses can be controlled or influenced through non-invasive techniques, especially those acting through vagal signaling mechanisms.

## Conclusion and future directions

The evidence across neuroscience, physiology, and sport science converges on one point: robust vagal tone is a reliable marker and modifiable driver of sharper cognition, steadier emotion, optimized arousal, and faster recovery in athletes. Simple, low-cost tools such as resonance-frequency breathing, HRVBFT, brief facial exposure to cold water, contemplative mindfulness practices, and transcutaneous VNS can enhance autonomic regulation and yield measurable gains, from improved executive function to higher VO_2_ max. Integrating these techniques with HRV-guided training loads and a polyvagal-informed team culture offers a pragmatic, non-pharmacological routes to enhancing *vagal agility*.

Wearable sensors and computational methods now provide continuous vagal biometrics and just-in-time coaching, while taVNS approaches offer the promise of on-demand neuromodulation and autonomic tuning. Research and validation can be further advanced by determining optimal taVNS frequency and dosing, evaluating individual moderators (baseline vagal tone, genotype, and training load), and studying seasonal and long-term outcomes. As adoption grows, safeguards around data privacy, informed consent, and balanced biomarker use are essential. The societal and ethical implications of utilizing neurotechnology for sports performance also needs to be given careful consideration. However, many natural top-down and bottom-up strategies discussed are globally available to everyone. These approaches should be implemented under the consultation of coaches, psychologists, and other performance experts to ensure athlete safety. Done properly with appropriate guidance or supervision, vagal-centric strategies can become a cornerstone of next-generation performance science. These approaches link lifestyle foundations to mental health while providing targeted interventions for broadening adaptive positive emotional responses, enhancing decision-making under pressure, and safeguarding the long-term resilience of athletes and other individuals seeking to optimize human performance and wellness.

## References

[B1] Ackermann S. P. Raab M. Backschat S. Smith D. J. C. Javelle F. Laborde S. (2022). The diving response and cardiac vagal activity: a systematic review and meta-analysis. Psychophysiology 60:e14183. 10.1111/psyp.14183 36219506

[B2] Ackland G. L. Patel A. B. U. Miller S. Gutierrez del Arroyo A. Thirugnanasambanthar J. Ravindran J. I.. (2025). Non-invasive vagus nerve stimulation and exercise capacity in healthy volunteers: a randomized trial. Eur. Heart J. 46, 1634–1644. 10.1093/eurheartj/ehaf037 39969124 PMC7617618

[B3] Afify A. M. (2023). Effect of diaphragmatic breathing exercise on cardiovascular parameters following noise exposure in pre hypertensive adults. J. Populat. Therap. Clin. Pharmacol. 30:10. 10.47750/jptcp.2023.30.07.010

[B4] Andersen H. T. (1963). The reflex nature of the physiological adjustments to diving and their afferent pathway. Acta Physiol. Scand. 58, 263–273. 10.1111/j.1748-1716.1963.tb02648.x 14012780

[B5] Anderson R. Hanrahan S. J. Mallett C. J. (2014). Investigating the optimal psychological state for peak performance in Australian elite athletes. J. Appl. Sport Psychol. 26, 318–333. 10.1080/10413200.2014.885915

[B6] Arakaki X. Arechavala R. J. Choy E. H. Bautista J. Bliss B. Molloy C.. (2023). The connection between heart rate variability (HRV), neurological health, and cognition: a literature review. Front. Neurosci. 17:1055445. 10.3389/fnins.2023.1055445 36937689 PMC10014754

[B7] Aston-Jones G. Cohen J. D. (2005). An integrative theory of locus coeruleus-norepinephrine function: adaptive gain and optimal performance. Annu. Rev. Neurosci. 28, 403–450. 10.1146/annurev.neuro.28.061604.135709 16022602

[B8] Aston-Jones G. Rajkowski J. Cohen J. (1999). Role of locus coeruleus in attention and behavioral flexibility. Biol. Psychiatry 46, 1309–1320. 10.1016/S0006-3223(99)00140-7 10560036

[B9] Bellenger C. R. Fuller J. T. Thomson R. L. Davison K. Robertson E. Y. Buckley J. D. (2016). Monitoring athletic training status through autonomic heart rate regulation: a systematic review and meta-analysis. Sports Med. 46, 1461–1486. 10.1007/s40279-016-0484-2 26888648

[B10] Berntson G. G. Cacioppo J. T. Quigley K. S. (1993). Respiratory sinus arrhythmia: Autonomic origins, physiological mechanisms, and psychophysiological implications. Psychophysiology 30, 183–196. 10.1111/j.1469-8986.1993.tb01731.x 8434081

[B11] Berthoud H.-R. Neuhuber W. L. (2000). Functional and chemical anatomy of the afferent vagal system. Auton. Neurosci. 85, 1–17. 10.1016/S1566-0702(00)00215-0 11189015

[B12] Borges U. Knops L. Laborde S. Klatt S. Raab M. (2020). Transcutaneous vagus nerve stimulation may enhance only specific aspects of the core executive functions. a randomized crossover trial. Front. Neurosci. 14:523. 10.3389/fnins.2020.00523 32523510 PMC7262369

[B13] Bretherton B. Atkinson L. Murray A. Clancy J. Deuchars S. Deuchars J. (2019). Effects of transcutaneous vagus nerve stimulation in individuals aged 55 years or above: potential benefits of daily stimulation. Aging 11, 4836–4857. 10.18632/aging.102074 31358702 PMC6682519

[B14] Butt M. F. Albusoda A. Farmer A. D. Aziz Q. (2019). The anatomical basis for transcutaneous auricular vagus nerve stimulation. J. Anat. 236, 588–611. 10.1111/joa.13122 31742681 PMC7083568

[B15] Çalι A. Özden A. V. Ceylan I. (2023). Effects of a single session of noninvasive auricular vagus nerve stimulation on sports performance in elite athletes: an open-label randomized controlled trial. Expert Rev. Med. Devices 21, 231–237. 10.1080/17434440.2023.2299300 38146234

[B16] Cansler R. Heidrich J. Whiting A. Tran D. Hall P. Tyler W. J. (2023). Influence of CrossFit and Deep End Fitness training on mental health and coping in athletes. Front. Sports Active Living 5:1061492. 10.3389/fspor.2023.1061492 37849685 PMC10577405

[B17] Carnevali L. Sgoifo A. (2014). Vagal modulation of resting heart rate in rats: the role of stress, psychosocial factors, and physical exercise. Front. Physiol. 5:118. 10.3389/fphys.2014.00118 24715877 PMC3970013

[B18] Chaitanya S. Datta A. Bhandari B. Sharma V. K. (2022). Effect of resonance breathing on heart rate variability and cognitive functions in young adults: a randomised controlled study. Cureus 14:e22187. 10.7759/cureus.22187 35308668 PMC8924557

[B19] Chen L. Tang C. Wang Z. Zhang L. Gu B. Liu X.. (2024). Enhancing motor sequence learning via transcutaneous auricular vagus nerve stimulation (taVNS): an EEG study. IEEE J. Biomed. Health Inform. 28, 1285–1296. 10.1109/JBHI.2023.3344176 38109248

[B20] Chen L. Zhang J. Wang Z. Zhang X. Zhang L. Xu M.. (2022). Effects of transcutaneous vagus nerve stimulation (tVNS) on action planning: a behavioural and EEG study. IEEE Trans. Neural Syst. Rehabilit. Eng. 30, 1675–1683. 10.1109/TNSRE.2021.3131497 34847035

[B21] Chen Y. Lu X. Hu L. (2023). Transcutaneous auricular vagus nerve stimulation facilitates cortical arousal and alertness. Int. J. Environ. Res. Public Health 20:1402. 10.3390/ijerph20021402 36674156 PMC9859411

[B22] Colzato L. S. Wolters G. Peifer C. (2017). Transcutaneous vagus nerve stimulation (tVNS) modulates flow experience. Exper. Brain Res. 236, 253–257. 10.1007/s00221-017-5123-0 29128975

[B23] Croft J. R. LaMacchia Z. M. Alderete J. F. Maestas A. Nguyen K. O'Hara R. B. (2025). Transcutaneous auricular vagus nerve stimulation: efficacy, applications, and challenges in mood disorders and autonomic regulation—a narrative review. Military Med. 21:usaf063. 10.1093/milmed/usaf063 40116526

[B24] Csikszentmihalyi M. (1988). “The flow experience and its significance for human psychology,” in Optimal Experience: Psychological Studies of Flow in Consciousness, eds. M. Csikszentmihalyi and I. S. Csikszentmihalyi (Cambridge: Cambridge University Press), 15–35. 10.1017/CBO9780511621956.002

[B25] Csikszentmihalyi M. (1990). Flow: The Psychology of Optimal Experience. New York: Harper and Row.

[B26] Czura C. J. Tracey K. J. (2005). Autonomic neural regulation of immunity. J. Intern. Med. 257, 156–166. 10.1111/j.1365-2796.2004.01442.x 15656874

[B27] de Souza P. M. Rosário N. S. A. de Castro Pinto K. M. Assunção P. E. de Oliveira F. L. P. Bearzoti E.. (2020). Vagal flexibility during exercise: impact of training, stress, anthropometric measures, and gender. Rehabil. Res. Pract. 2020, 1–8. 10.1155/2020/6387839 33083060 PMC7556060

[B28] Dergacheva O. Griffioen K. J. Neff R. A. Mendelowitz D. (2010). Respiratory modulation of premotor cardiac vagal neurons in the brainstem. Respir. Physiol. Neurobiol. 174, 102–110. 10.1016/j.resp.2010.05.005 20452467 PMC2932818

[B29] Faller J. Cummings J. Saproo S. Sajda P. (2019). Regulation of arousal via online neurofeedback improves human performance in a demanding sensory-motor task. Proc. Nat. Acad. Sci. 116, 6482–6490. 10.1073/pnas.1817207116 30862731 PMC6442591

[B30] Fisher A. J. Song J. Soyster P. D. (2021). Toward a systems-based approach to understanding the role of the sympathetic nervous system in depression. World Psychiatry 20, 295–296. 10.1002/wps.20872 34002517 PMC8129862

[B31] Forte G. Casagrande M. (2025). The intricate brain–heart connection: the relationship between heart rate variability and cognitive functioning. Neuroscience 565, 369–376. 10.1016/j.neuroscience.2024.12.004 39645073

[B32] Frangos E. Ellrich J. Komisaruk B. R. (2015). Non-invasive access to the vagus nerve central projections via electrical stimulation of the external ear: fMRI evidence in humans. Brain Stimul. 8, 624–636. 10.1016/j.brs.2014.11.018 25573069 PMC4458242

[B33] Gamaiunova L. Brandt P.-Y. Bondolfi G. Kliegel M. (2019). Exploration of psychological mechanisms of the reduced stress response in long-term meditation practitioners. Psychoneuroendocrinology 104, 143–151. 10.1016/j.psyneuen.2019.02.026 30849720

[B34] Garcia R. G. Cohen J. E. Stanford A. D. Gabriel A. Stowell J. Aizley H.. (2021). Respiratory-gated auricular vagal afferent nerve stimulation (RAVANS) modulates brain response to stress in major depression. J. Psychiatr. Res. 142, 188–197. 10.1016/j.jpsychires.2021.07.048 34365067 PMC8429271

[B35] Garcia R. G. Staley R. Aroner S. Stowell J. Sclocco R. Napadow V.. (2022). Optimization of respiratory-gated auricular vagus afferent nerve stimulation for the modulation of blood pressure in hypertension. Front. Neurosci. 16:1038339. 10.3389/fnins.2022.1038339 36570845 PMC9783922

[B36] Geisler F. C. M. Kubiak T. Siewert K. Weber H. (2013). Cardiac vagal tone is associated with social engagement and self-regulation. Biol. Psychol. 93, 279–286. 10.1016/j.biopsycho.2013.02.013 23466587

[B37] Gerritsen R. J. S. Band G. P. H. (2018). Breath of life: the respiratory vagal stimulation model of contemplative activity. Front. Hum. Neurosci. 12:397. 10.3389/fnhum.2018.00397 30356789 PMC6189422

[B38] Goessl V. C. Curtiss J. E. Hofmann S. G. (2017). The effect of heart rate variability biofeedback training on stress and anxiety: a meta-analysis. Psychol. Med. 47, 2578–2586. 10.1017/S0033291717001003 28478782

[B39] Gooden B. A. (1994). Mechanism of the human diving response. Integr. Physiol. Behav. Sci. 29, 6–16. 10.1007/BF02691277 8018553

[B40] Gourine A. V. Ackland G. L. (2019). Cardiac vagus and exercise. Physiology. 34, 71–80. 10.1152/physiol.00041.2018 30540229 PMC6383634

[B41] Gullett N. Zajkowska Z. Walsh A. Harper R. Mondelli V. (2023). Heart rate variability (HRV) as a way to understand associations between the autonomic nervous system (ANS) and affective states: a critical review of the literature. Int. J. Psychophysiol. 192, 35–42. 10.1016/j.ijpsycho.2023.08.001 37543289

[B42] Gurel N. Z. Wittbrodt M. T. Jung H. Shandhi M.d. M. H Driggers E. G.. (2020). Transcutaneous cervical vagal nerve stimulation reduces sympathetic responses to stress in posttraumatic stress disorder: a double-blind, randomized, sham controlled trial. Neurobiol. Stress 13:100264. 10.1016/j.ynstr.2020.100264 33344717 PMC7739181

[B43] Handforth A. DeGiorgio C. M. Schachter S. C. Uthman B. M. Naritoku D. K. Tecoma E. S.. (1998). Vagus nerve stimulation therapy for partial-onset seizures. Neurology 51, 48–55. 10.1212/WNL.51.1.48 9674777

[B44] Hansen A. L. Johnsen B. H. Thayer J. F. (2003). Vagal influence on working memory and attention. Int. J. Psychophysiol. 48, 263–274. 10.1016/S0167-8760(03)00073-4 12798986

[B45] Harris D. J. Allen K. L. Vine S. J. Wilson M. R. (2021). A systematic review and meta-analysis of the relationship between flow states and performance. Int. Rev. Sport Exerc. Psychol. 16, 693–721. 10.1080/1750984X.2021.1929402

[B46] Hatik S. H. Arslan M. Demirbilek Ö. Özden A. V. (2023). The effect of transcutaneous auricular vagus nerve stimulation on cycling ergometry and recovery in healthy young individuals. Brain Behav. 13:e3332. 10.1002/brb3.3332 37974551 PMC10726880

[B47] Henriques G. Keffer S. Abrahamson C. Jeanne Horst S. (2011). Exploring the effectiveness of a computer-based heart rate variability biofeedback program in reducing anxiety in college students. Appl. Psychophysiol. Biofeedback 36, 101–112. 10.1007/s10484-011-9151-4 21533678

[B48] Hurwitz B. E. Furedy J. J. (1986). The human dive reflex: an experimental, topographical and physiological analysis. Physiol. Behav. 36, 287–294. 10.1016/0031-9384(86)90018-1 3961003

[B49] Jacobs H. I. L. Riphagen J. M. Razat C. M. Wiese S. Sack A. T. (2015). Transcutaneous vagus nerve stimulation boosts associative memory in older individuals. Neurobiol. Aging 36, 1860–1867. 10.1016/j.neurobiolaging.2015.02.023 25805212

[B50] Jigo M. Carmel J. B. Wang Q. Rodenkirch C. (2024). Transcutaneous cervical vagus nerve stimulation improves sensory performance in humans: a randomized controlled crossover pilot study. Sci. Rep. 14:3975. 10.1038/s41598-024-54026-8 38368486 PMC10874458

[B51] Jiménez Morgan S. Molina Mora J. A. (2017). Effect of heart rate variability biofeedback on sport performance, a systematic review. Appl. Psychophysiol. Biofeedback 42, 235–245. 10.1007/s10484-017-9364-2 28573597

[B52] Jongkees B. J. Immink M. A. Finisguerra A. Colzato L. S. (2018). Transcutaneous vagus nerve stimulation (tVNS) enhances response selection during sequential action. Front. Psychol. 9:1159. 10.3389/fpsyg.2018.01159 30034357 PMC6043681

[B53] Karavidas M. K. Lehrer P. M. Vaschillo E. Vaschillo B. Marin H. Buyske S.. (2007). Preliminary results of an open label study of heart rate variability biofeedback for the treatment of major depression. Appl. Psychophysiol. Biofeedback 32, 19–30. 10.1007/s10484-006-9029-z 17333315

[B54] Kelly M. J. Breathnach C. Tracey K. J. Donnelly S. C. (2022). Manipulation of the inflammatory reflex as a therapeutic strategy. Cell Rep. Med. 3:100696. 10.1016/j.xcrm.2022.100696 35858588 PMC9381415

[B55] Khurana R. K. Watabiki S. Hebel J. R. Toro R. Nelson E. (1980). Cold face test in the assessment of trigeminal-brainstem- vagal function in humans. Ann. Neurol. 7, 144–149. 10.1002/ana.410070209 7369721

[B56] Kim A. Y. Marduy A. de Melo P. S. Gianlorenco A. C. Kim C. K. Choi H.. (2022). Safety of transcutaneous auricular vagus nerve stimulation (taVNS): a systematic review and meta-analysis. Sci. Rep. 12:22055. 10.1038/s41598-022-25864-1 36543841 PMC9772204

[B57] Kinoshita T. Nagata S. Baba R. Kohmoto T. Iwagaki S. (2006). Cold-water face immersion per se elicits cardiac parasympathetic activity. Circ. J. 70, 773–776. 10.1253/circj.70.773 16723802

[B58] Kiviniemi A. M. Hautala A. J. Kinnunen H. Tulppo M. P. (2007). Endurance training guided individually by daily heart rate variability measurements. Eur. J. Appl. Physiol. 101, 743–751. 10.1007/s00421-007-0552-2 17849143

[B59] Kraus T. Hösl K. Kiess O. Schanze A. Kornhuber J. Forster C. (2007). BOLD fMRI deactivation of limbic and temporal brain structures and mood enhancing effect by transcutaneous vagus nerve stimulation. J. Neural Transm. 114, 1485–1493. 10.1007/s00702-007-0755-z 17564758

[B60] Krygier J. R. Heathers J. A. J. Shahrestani S. Abbott M. Gross J. J. Kemp A. H. (2013). Mindfulness meditation, well-being, and heart rate variability: A preliminary investigation into the impact of intensive Vipassana meditation. Int. J. Psychophysiol. 89, 305–313. 10.1016/j.ijpsycho.2013.06.017 23797150

[B61] Laborde S. Mosley E. Mertgen A. (2018a). Vagal tank theory: the three RS of cardiac vagal control functioning – resting, reactivity, and recovery. Front. Neurosci. 12:458. 10.3389/fnins.2018.00458 30042653 PMC6048243

[B62] Laborde S. Mosley E. Thayer J. F. (2017). Heart rate variability and cardiac vagal tone in psychophysiological research – recommendations for experiment planning, data analysis, and data reporting. Front. Psychol. 08:213. 10.3389/fpsyg.2017.00213 28265249 PMC5316555

[B63] Laborde S. Mosley E. Ueberholz L. (2018b). Enhancing cardiac vagal activity: factors of interest for sport psychology. Sport Brain 240, 71–92. 10.1016/bs.pbr.2018.09.002 30390842

[B64] Lagos L. Vaschillo E. Vaschillo B. Lehrer P. Bates M. Pandina R. (2008). Heart Rate Variability Biofeedback as a Strategy for Dealing with Competitive Anxiety: A Case Study. New York: Academic press.

[B65] Langdeau J.-B. Turcotte H. Desgagné P. Jobin J. Boulet L.-P. (2000). Influence of sympatho-vagal balance on airway responsiveness in athletes. Eur. J. Appl. Physiol. 83, 370–375. 10.1007/s004210000306 11138577

[B66] Le Meur Y. Pichon A. Schaal K. Schmitt L. Louis J. Gueneron J.. (2013). Evidence of parasympathetic hyperactivity in functionally overreached athletes. Med. Sci. Sports Exerc. 45, 2061–2071. 10.1249/MSS.0b013e3182980125 24136138

[B67] Lee J. Kim J. K. Wachholtz A. (2015). The benefit of heart rate variability biofeedback and relaxation training in reducing trait anxiety. Hanguk Simni Hakhoe Chi Kongang 20, 391–408. 10.17315/kjhp.2015.20.2.002 27099546 PMC4835037

[B68] Lehrer P. M. Gevirtz R. (2014). Heart rate variability biofeedback: how and why does it work? Front. Psychol. 5:756. 10.3389/fpsyg.2014.00756 25101026 PMC4104929

[B69] Lehrer P. M. Vaschillo E. Vaschillo B. Lu S.-E. Eckberg D. L. Edelberg R.. (2003). Heart rate variability biofeedback increases baroreflex gain and peak expiratory flow. Psychosom. Med. 65, 796–805. 10.1097/01.PSY.0000089200.81962.19 14508023

[B70] Liu C.-H. Yang M.-H. Zhang G.-Z. Wang X.-X. Li B. Li M.. (2020). Neural networks and the anti-inflammatory effect of transcutaneous auricular vagus nerve stimulation in depression. J. Neuroinflam. 17:54. 10.1186/s12974-020-01732-5 32050990 PMC7017619

[B71] Lumma A.-L. Kok B. E. Singer T. (2015). Is meditation always relaxing? Investigating heart rate, heart rate variability, experienced effort and likeability during training of three types of meditation. Int. J. Psychophysiol. 97, 38–45. 10.1016/j.ijpsycho.2015.04.017 25937346

[B72] Machetanz K. Berelidze L. Guggenberger R. Gharabaghi A. (2021a). Transcutaneous auricular vagus nerve stimulation and heart rate variability: analysis of parameters and targets. Auton. Neurosci. 236:102894. 10.1016/j.autneu.2021.102894 34662844

[B73] Machetanz K. Berelidze L. Guggenberger R. Gharabaghi A. (2021b). Brain–heart interaction during transcutaneous auricular vagus nerve stimulation. Front. Neurosci. 15:632697. 10.3389/fnins.2021.632697 33790736 PMC8005577

[B74] Malik M. (1996). Heart rate variability. Ann. Noninv. Electrocardiol. 1, 151–181. 10.1111/j.1542-474X.1996.tb00275.x

[B75] Mao Y. Chen C. Falahpour M. MacNiven K. H. Heit G. Sharma V.. (2022). Effects of sub-threshold transcutaneous auricular vagus nerve stimulation on cingulate cortex and insula resting-state functional connectivity. Front. Hum. Neurosci. 16:862443. 10.3389/fnhum.2022.862443 35496068 PMC9048677

[B76] McCraty R. (2017). New frontiers in heart rate variability and social coherence research: techniques, technologies, and implications for improving group dynamics and outcomes. Front. Public Health 5:267. 10.3389/fpubh.2017.00267 29075623 PMC5643505

[B77] McLaughlin K. A. Rith-Najarian L. Dirks M. A. Sheridan M. A. (2013). Low vagal tone magnifies the association between psychosocial stress exposure and internalizing psychopathology in adolescents. J. Clin. Child Adoles. Psychol. 44, 314–328. 10.1080/15374416.2013.843464 24156380 PMC4076387

[B78] Meeusen R. Duclos M. Foster C. Fry A. Gleeson M. Nieman D.. (2013). Prevention, diagnosis, and treatment of the overtraining syndrome: joint consensus statement of the European College of Sport Science and the American College of Sports Medicine. Med. Sci. Sports Exerc. 45, 186–205. 10.1249/MSS.0b013e318279a10a 23247672

[B79] Miyatsu T. Oviedo V. Reynaga J. Karuzis V. P. Martinez D. O'Rourke P.. (2024). Transcutaneous cervical vagus nerve stimulation enhances second-language vocabulary acquisition while simultaneously mitigating fatigue and promoting focus. Sci. Rep. 14:17177. 10.1038/s41598-024-68015-4 39060415 PMC11282064

[B80] Moazzami K. Pearce B. D. Gurel N. Z. Wittbrodt M. T. Levantsevych O. M. Huang M.. (2023). Transcutaneous vagal nerve stimulation modulates stress-induced plasma ghrelin levels: A double-blind, randomized, sham-controlled trial. J. Affect. Disord. 342, 85–90. 10.1016/j.jad.2023.09.015 37714385 PMC10698687

[B81] Mosley E. Laborde S. Kavanagh E. (2017). The contribution of coping related variables and cardiac vagal activity on the performance of a dart throwing task under pressure. Physiol. Behav. 179, 116–125. 10.1016/j.physbeh.2017.05.030 28577887

[B82] Murphy A. J. O'Neal A. G. Cohen R. A. Lamb D. G. Porges E. C. Bottari S. A.. (2023). The effects of transcutaneous vagus nerve stimulation on functional connectivity within semantic and hippocampal networks in mild cognitive impairment. Neurotherapeutics 20, 419–430. 10.1007/s13311-022-01318-4 36477709 PMC10121945

[B83] Noakes T. D. St Clair Gibson A. Lambert E. V. (2005). From catastrophe to complexity: a novel model of integrative central neural regulation of effort and fatigue during exercise in humans: summary and conclusions. Br. J. Sports Med. 39, 120–124. 10.1136/bjsm.2003.010330 15665213 PMC1725112

[B84] Olsen L. K. Solis E. McIntire L. K. Hatcher-Solis C. N. (2023). Vagus nerve stimulation: mechanisms and factors involved in memory enhancement. Front. Hum. Neurosci. 17:1152064. 10.3389/fnhum.2023.1152064 37457500 PMC10342206

[B85] Ortega E. Wang C. J. K. (2017). Pre-performance physiological state: heart rate variability as a predictor of shooting performance. Appl. Psychophysiol. Biofeedback 43, 75–85. 10.1007/s10484-017-9386-9 29124507

[B86] Paciorek A. Skora L. (2020). Vagus nerve stimulation as a gateway to interoception. Front. Psychol. 11:1659. 10.3389/fpsyg.2020.01659 32849014 PMC7403209

[B87] Pandža N. B. Phillips I. Karuzis V. P. O'Rourke P. Kuchinsky S. E. (2020). Neurostimulation and pupillometry: new directions for learning and research in applied linguistics. Annu. Rev. Appl. Linguist. 40, 56–77. 10.1017/S026719052000006934673939

[B88] Pavlov V. A. Tracey K. J. (2012). The vagus nerve and the inflammatory reflex—linking immunity and metabolism. Nat. Rev. Endocrinol. 8, 743–754. 10.1038/nrendo.2012.189 23169440 PMC4082307

[B89] Pavlov V. A. Tracey K. J. (2022). Bioelectronic medicine: preclinical insights and clinical advances. Neuron 110, 3627–3644. 10.1016/j.neuron.2022.09.003 36174571 PMC10155266

[B90] Peifer C. Schulz A. Schächinger H. Baumann N. Antoni C. H. (2014). The relation of flow-experience and physiological arousal under stress—Can u shape it? J. Exp. Soc. Psychol. 53, 62–69. 10.1016/j.jesp.2014.01.009

[B91] Phillips I. Calloway R. C. Karuzis V. P. PandŽa N. B. O'Rourke P. Kuchinsky S. E. (2021). Transcutaneous auricular vagus nerve stimulation strengthens semantic representations of foreign language tone words during initial stages of learning. J. Cogn. Neurosci. 34, 127–152. 10.1162/jocn_a_01783 34673939

[B92] Phillips I. Johns M. A. PandŽa N. B. Calloway R. C. Karuzis V. P. Kuchinsky S. E. (2025). Three hundred hertz transcutaneous auricular vagus nerve stimulation (taVNS) impacts pupil size non-linearly as a function of intensity. Psychophysiology 62:70011. 10.1111/psyp.70011 40013407 PMC11866280

[B93] Plews D. J. Laursen P. B. Stanley J. Kilding A. E. Buchheit M. (2013). Training adaptation and heart rate variability in elite endurance athletes: opening the door to effective monitoring. Sports Med. 43, 773–781. 10.1007/s40279-013-0071-8 23852425

[B94] Porges S. W. (2001). The polyvagal theory: phylogenetic substrates of a social nervous system. Int. J. Psychophysiol. 42, 123–146. 10.1016/S0167-8760(01)00162-3 11587772

[B95] Porges S. W. (2007). The polyvagal perspective. Biol. Psychol. 74, 116–143. 10.1016/j.biopsycho.2006.06.009 17049418 PMC1868418

[B96] Porges S. W. (2009). The polyvagal theory: new insights into adaptive reactions of the autonomic nervous system. Cleveland Clinic J. Med. 76, S86–S90. 10.3949/ccjm.76.s2.17 19376991 PMC3108032

[B97] Rufener K. S. Geyer U. Janitzky K. Heinze H. Zaehle T. (2018). Modulating auditory selective attention by non-invasive brain stimulation: differential effects of transcutaneous vagal nerve stimulation and transcranial random noise stimulation. Eur. J. Neurosci. 48, 2301–2309. 10.1111/ejn.14128 30144194

[B98] Schipke J. D. Pelzer M. (2001). Effect of immersion, submersion, and scuba diving on heart rate variability: figure 1. Br. J. Sports Med. 35, 174–180. 10.1136/bjsm.35.3.174 11375876 PMC1724326

[B99] Sclocco R. Garcia R. G. Kettner N. W. Isenburg K. Fisher H. P. Hubbard C. S.. (2019). The influence of respiration on brainstem and cardiovagal response to auricular vagus nerve stimulation: a multimodal ultrahigh-field (7T) fMRI study. Brain Stimul. 12, 911–921. 10.1016/j.brs.2019.02.003 30803865 PMC6592731

[B100] Segerstrom S. C. Nes L. S. (2007). Heart rate variability reflects self-regulatory strength, effort, and fatigue. Psychol. Sci. 18, 275–281. 10.1111/j.1467-9280.2007.01888.x 17444926

[B101] Shaffer F. Meehan Z. M. (2020). A practical guide to resonance frequency assessment for heart rate variability biofeedback. Front. Neurosci. 14:570400. 10.3389/fnins.2020.570400 33117119 PMC7578229

[B102] Sharon O. Fahoum F. Nir Y. (2020). Transcutaneous vagus nerve stimulation in humans induces pupil dilation and attenuates alpha oscillations. J. Neurosci. 41, 320–330. 10.1523/JNEUROSCI.1361-20.2020 33214317 PMC7810665

[B103] Silberstein S. D. Mechtler L. L. Kudrow D. B. Calhoun A. H. McClure C. Saper J. R.. (2016). Non–invasive vagus nerve stimulation for the ACute treatment of cluster headache: findings from the randomized, double-blind, sham-controlled ACT1 study. Headache 56, 1317–1332. 10.1111/head.12896 27593728 PMC5113831

[B104] Sloan R. P. Shapiro P. A. Bagiella E. Boni S. M. Paik M. Bigger J. T.. (1994). Effect of mental stress throughout the day on cardiac autonomic control. Biol. Psychol. 37, 89–99. 10.1016/0301-0511(94)90024-8 8003592

[B105] Sommer A. Fischer R. Borges U. Laborde S. Achtzehn S. Liepelt R. (2023). The effect of transcutaneous auricular vagus nerve stimulation (taVNS) on cognitive control in multitasking. Neuropsychologia 187:108614. 10.1016/j.neuropsychologia.2023.108614 37295553

[B106] Steffen P. R. Austin T. DeBarros A. Brown T. (2017). The impact of resonance frequency breathing on measures of heart rate variability, blood pressure, and mood. Front. Public Health 5:222. 10.3389/fpubh.2017.00222 28890890 PMC5575449

[B107] Sun J.-B. Cheng C. Tian Q.-Q. Yuan H. Yang X.-J. Deng H.. (2021). Transcutaneous auricular vagus nerve stimulation improves spatial working memory in healthy young adults. Front. Neurosci. 15:790793. 10.3389/fnins.2021.790793 35002607 PMC8733384

[B108] Szeska C. Klepzig K. Hamm A. O. Weymar M. (2025). Ready for translation: non-invasive auricular vagus nerve stimulation inhibits psychophysiological indices of stimulus-specific fear and facilitates responding to repeated exposure in phobic individuals. Transl. Psychiatry 15:135. 10.1038/s41398-025-03352-0 40204704 PMC11982236

[B109] Szulczewski M. T. (2022). Transcutaneous auricular vagus nerve stimulation combined with slow breathing: speculations on potential applications and technical considerations. Neuromodulation 25, 380–394. 10.1111/ner.13458 35396070

[B110] Szulczewski M. T. D'Agostini M. Van Diest I. (2023). Expiratory-gated transcutaneous auricular vagus nerve stimulation (taVNS) does not further augment heart rate variability during slow breathing at 0.1 Hz. Appl. Psychophysiol. Biofeedback 48, 323–333. 10.1007/s10484-023-09584-4 36920567 PMC10412484

[B111] Taublieb P. (2018). “Mind Gurus,” in Enhanced, Taublieb Films and Jigsaw Productions for ESPN Films. Available online at: https://vimeo.com/281118327 (Accessed June 1, 2025).

[B112] Thayer J. F. Yamamoto S. S. Brosschot J. F. (2010). The relationship of autonomic imbalance, heart rate variability and cardiovascular disease risk factors. Int. J. Cardiol. 141, 122–131. 10.1016/j.ijcard.2009.09.543 19910061

[B113] Trifilio E. Shortell D. Olshan S. O'Neal A. Coyne J. Lamb D.. (2023). Impact of transcutaneous vagus nerve stimulation on healthy cognitive and brain aging. Front. Neurosci. 17:1184051. 10.3389/fnins.2023.1184051 37575296 PMC10416636

[B114] Tyler W. J. (2025). Auricular bioelectronic devices for health, medicine, and human-computer interfaces. Front. Electr. 6:1503425. 10.3389/felec.2025.1503425

[B115] Tyler W. J. Adavikottu A. Blanco C. L. Mysore A. Blais C. Santello M.. (2025). Neurotechnology for enhancing human operation of robotic and semi-autonomous systems. Front. Robot. AI 12:1491494. 10.3389/frobt.2025.1491494 40485770 PMC12141011

[B116] Tyler W. J. Boasso A. M. Mortimore H. M. Silva R. S. Charlesworth J. D. Marlin M. A.. (2015). Transdermal neuromodulation of noradrenergic activity suppresses psychophysiological and biochemical stress responses in humans. Sci. Rep. 5:e13865. 10.1038/srep13865 26353920 PMC4564766

[B117] Tyler W. J. Wyckoff S. Hearn T. Hool N. (2019). The safety and efficacy of transdermal auricular vagal nerve stimulation earbud electrodes for modulating autonomic arousal, attention, sensory gating, and cortical brain plasticity in humans. bioRxiv, 732529. 10.1101/732529

[B118] Urbin M. A. Lafe C. W. Simpson T. W. Wittenberg G. F. Chandrasekaran B. Weber D. J. (2021). Electrical stimulation of the external ear acutely activates noradrenergic mechanisms in humans. Brain Stimul. 14, 990–1001. 10.1016/j.brs.2021.06.002 34154980

[B119] van der Linden D. Tops M. Bakker A. B. (2021). The neuroscience of the flow state: involvement of the locus coeruleus norepinephrine system. Front. Psychol. 12:645498. 10.3389/fpsyg.2021.645498 33935902 PMC8079660

[B120] van der Zwan J. E. de Vente W. Huizink A. C. Bögels S. M. de Bruin E. I. (2015). Physical activity, mindfulness meditation, or heart rate variability biofeedback for stress reduction: a randomized controlled trial. Appl. Psychophysiol. Biofeedback 40, 257–268. 10.1007/s10484-015-9293-x 26111942 PMC4648965

[B121] Van Diest I. Verstappen K. Aubert A. E. Widjaja D. Vansteenwegen D. Vlemincx E. (2014). Inhalation/exhalation ratio modulates the effect of slow breathing on heart rate variability and relaxation. Appl. Psychophysiol. Biofeedback 39, 171–180. 10.1007/s10484-014-9253-x 25156003

[B122] Vaschillo E. Lehrer P. Rishe N. Konstantinov M. (2002). Heart rate variability biofeedback as a method for assessing baroreflex function: a preliminary study of resonance in the cardiovascular system. Appl. Psychophysiol. Biofeedback 27, 1–27. 10.1023/A:1014587304314 12001882

[B123] Vesterinen V. Nummela A. Heikura I. Laine T. Hynynen E. Botella J.. (2016). Individual endurance training prescription with heart rate variability. Med. Sci. SportsExer. 48, 1347–1354. 10.1249/MSS.0000000000000910 26909534

[B124] Villani V. Tsakiris M. Azevedo R. T. (2019). Transcutaneous vagus nerve stimulation improves interoceptive accuracy. Neuropsychologia 134:107201. 10.1016/j.neuropsychologia.2019.107201 31562863

[B125] Wei L. Chen Y. Chen X. Baeken C. Wu G.-R. (2024). Cardiac vagal activity changes moderated the association of cognitive and cerebral hemodynamic variations in the prefrontal cortex. Neuroimage 297:120725. 10.1016/j.neuroimage.2024.120725 38977040

[B126] Wells R. Outhred T. Heathers J. A. J. Quintana D. S. Kemp A. H. (2012). Matter over mind: a randomised-controlled trial of single-session biofeedback training on performance anxiety and heart rate variability in musicians. PLoS ONE 7:e46597. 10.1371/journal.pone.0046597 23056361 PMC3464298

[B127] Yap J. Y. Y. Keatch C. Lambert E. Woods W. Stoddart P. R. Kameneva T. (2020). Critical review of transcutaneous vagus nerve stimulation: challenges for translation to clinical practice. Front. Neurosci. 14:284. 10.3389/fnins.2020.00284 32410932 PMC7199464

[B128] Yerkes R. M. Dodson J. D. (1908). The relation of strength of stimulus to rapidity of habit-formation. J. Compar. Neurol. Psychol. 18, 459–482. 10.1002/cne.920180503

[B129] Yoshida Y. Okayama S. Fujihara D. Taniyama M. Yamada A. Fukui M.. (2025). Effects of transcutaneous auricular vagus nerve stimulation on hemodynamics and autonomic function during exercise stress tests in healthy volunteers. Circul. Rep. 7, 315–322. 10.1253/circrep.CR-24-0136 40352124 PMC12061502

[B130] Yuan H. Silberstein S. D. (2015). Vagus nerve and vagus nerve stimulation, a comprehensive review: part I. Headache 56, 71–78. 10.1111/head.12647 26364692

[B131] Zagon A. (2001). Does the vagus nerve mediate the sixth sense? Trends Neurosci. 24, 671–673. 10.1016/S0166-2236(00)01929-9 11672813

[B132] Zhao Q. Yu C. D. Wang R. Xu Q. J. Dai Pra R. Zhang L.. (2022). A multidimensional coding architecture of the vagal interoceptive system. Nature 603, 878–884. 10.1038/s41586-022-04515-5 35296859 PMC8967724

